# Case report: Catheter ablation for persistent atrial fibrillation in a patient with heart of stone

**DOI:** 10.3389/fcvm.2023.1207064

**Published:** 2023-10-02

**Authors:** Yingjian Deng, Guiyang Li, Jianghai Liu, Xingcai Wan, Linlin Li, Jialan Lv, Qiang Li, Faguang Zhou, Dong Chang

**Affiliations:** Department of Cardiology, Xiamen Cardiovascular Hospital of Xiamen University, School of Medicine, Xiamen University, Xiamen, China

**Keywords:** myocardial calcification, arrhythmia, atrial fibrillation, catheter ablation, case report

## Abstract

Myocardial calcification is a rare condition, with only a few reports in the literature. For the first time, we report a case of diffuse myocardial calcification who underwent successful catheter ablation for persistent atrial fibrillation (AF). In this case, catheter ablation was recommended due to repeated hospitalization for palpitation and heart failure, but preoperative computed tomography showed massive myocardial calcification. Electroanatomic mapping of the atrium was performed with a Pentaray catheter before ablation, which showed areas of low voltage in the calcified region. As the persistent AF was terminated after circumferential pulmonary vein isolation and posterior wall isolation, and no further ablation was performed. The patient recovered well, with no recurrence of palpitation or heart failure during the one-year follow-up.

## 1. Introduction

Myocardial calcification is categorized as either dystrophic or metastatic calcification in the myocardium and is associated with cardiac dysfunction and poor prognosis (1). Dystrophic calcification occurs most commonly after myocardial infarction, whereas metastatic calcification is usually associated with abnormalities of calcium homeostasis, such as chronic renal failure, hyperparathyroidism, and bone diseases (2, 3). Previous reports have suggested that calcium deposition can disturb the cardiac conduction system, leading to cardiac arrhythmias or sudden death (4). Catheter ablation is an effective and well-established treatment for atrial fibrillation (AF); however, there have been no previous reports of catheter ablation in patients with myocardial calcification, resulting in a lack of clinical evidence. For the first time, we report a case of diffuse myocardial calcification in a patient who underwent successful catheter ablation for persistent AF.

## 2. Case presentation

A 56-year-old male with hypertension, recurrent palpitations, and shortness of breath for over 5 years, was referred to our hospital for further evaluation and treatment, as the electrocardiogram showed persistent AF. Over 10 years ago, the patient was diagnosed with myocarditis. Recently, he was repeatedly hospitalized for tachycardia-induced heart failure and showed no noticeable improvement after pharmacotherapy.

**Figure 1:**
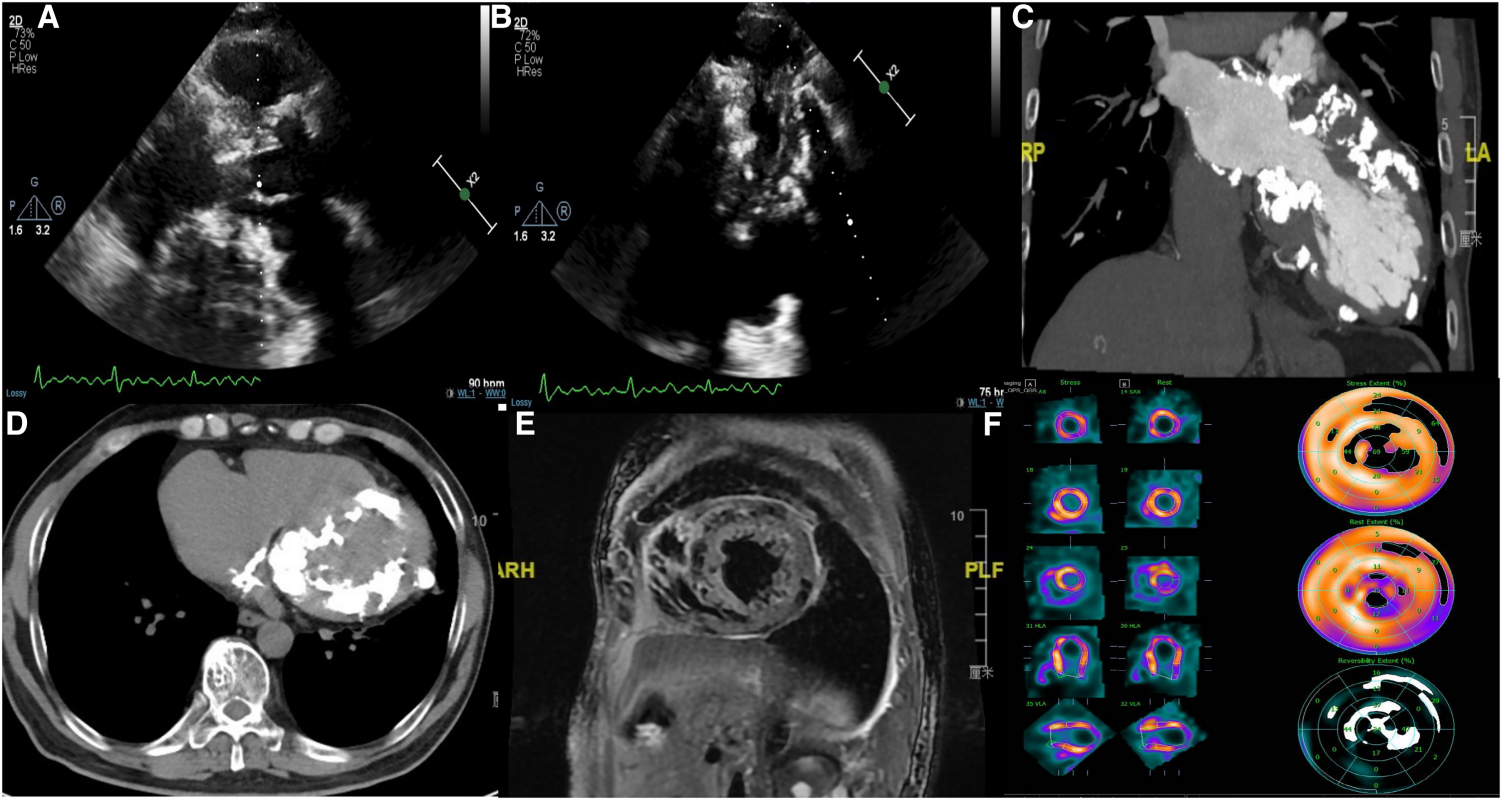
(**A,B**) Transthoracic echocardiography showing interventricular septal hypertrophy, extensive calcification in the left atrial wall, left ventricle, mitral annulus, and aortic wall; (**C,D**) cardiac computed tomography showing extensive calcification in the left ventricle, left atrium, atrial septa, and mitral annulus; (**E**) cardiac magnetic resonance imaging showing diffuse myocardial calcification; (**F**) radionuclide myocardial perfusion imaging (stress/rest) showing fixed perfusion defects.

**Figure 2:**
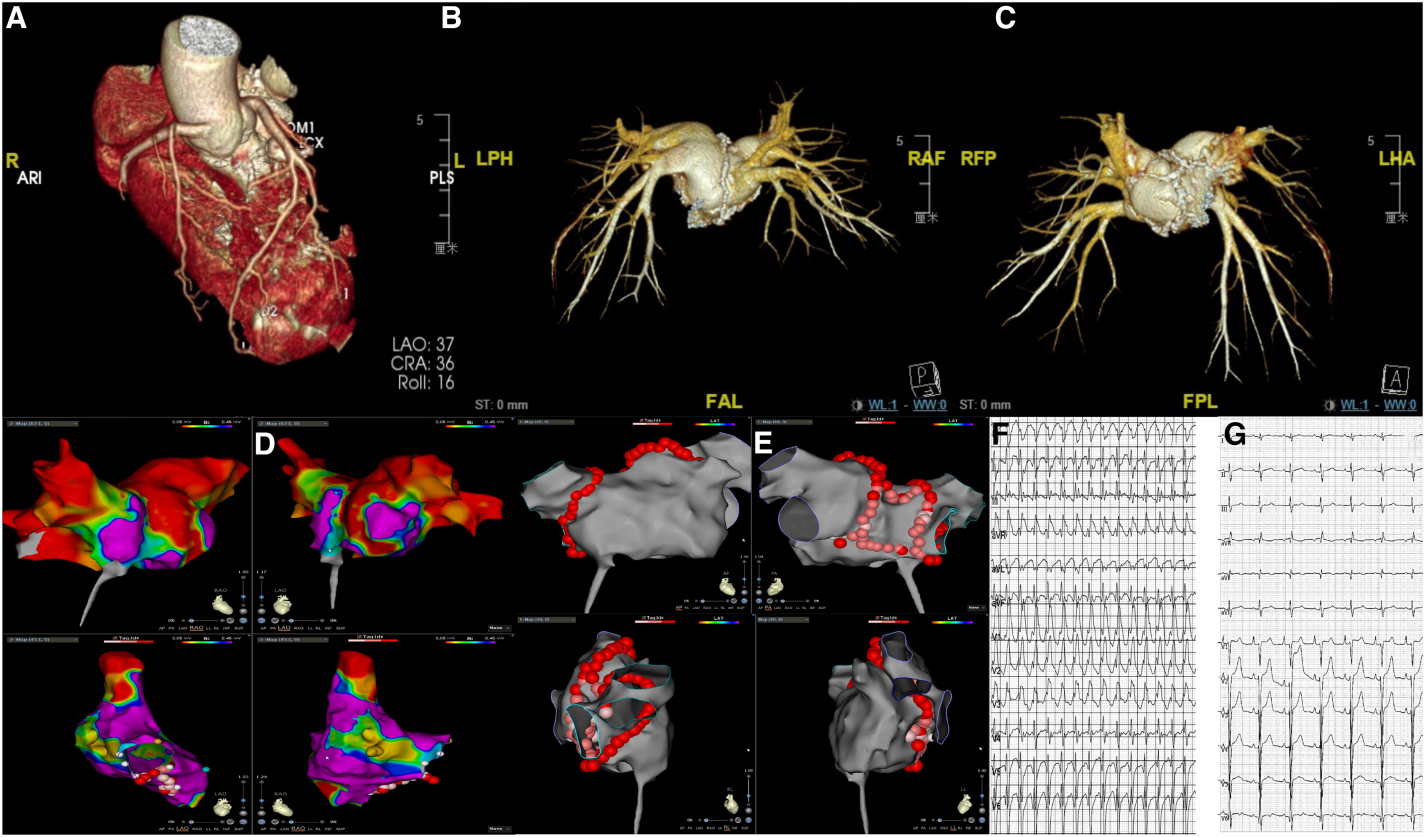
(**A**) Coronary computed tomography angiography showing no significant coronary stenosis; (**B,C**) the 3-dimensional reconstruction of the left atrium and pulmonary veins showing calcification in the left atrium; (**D**) three-dimensional voltage map of the left and right atrium showing areas of low voltage; (**E**) ablation lesion sets (red dots) of pulmonary vein isolation and posterior left atrial wall isolation; (**F**) preoperative 12-lead ECG showing atrial fibrillation with a rapid ventricular rate; (**G**) postoperative 12-lead ECG showing sinus rhythm.

Transthoracic echocardiography showed diffuse myocardial thickening (septal thickness 15.2 mm) and calcification (mainly involving the left atrial wall, left ventricle, atrial septa, mitral annulus, and aortic wall) (Figures 1A, B). Cardiac computed tomography and magnetic resonance imaging also indicated extensive calciﬁcations (Figures 1C–E). Both echocardiography and cardiac magnetic resonance imaging suggested a significant decrease in systolic and diastolic function, but the severe calcification of the myocardium and mitral annulus made the measurement difficult. Radiographic myocardial perfusion imaging (Stress/Rest) showed fixed perfusion defects (Figure 1F). No significant coronary stenosis was detected on coronary computed tomography angiography (Figure 2A). The 3-dimensional reconstruction of the left atrium and pulmonary veins is presented in Figures 2B, C. His laboratory tests showed that N-terminal pro-B-type natriuretic peptide was 2,952 pg/ml (0–125 pg/ml), calcium was 2.32 mmol/L (2.11–2.52 mmol/L), and phosphorus was 0.7 mmol/L (0.85–1.51 mmol/L). The patient had no other relevant abnormalities in hematology or biochemistry, including renal and liver function, thyroid function, calcium metabolism, autoimmune function, and inflammatory indicators. He has no history of previous myocardial infarction or tuberculosis. Therefore, we speculated that myocardial calcification may have developed secondary to myocarditis. However, detailed information on the previous history of myocarditis was missing.

Despite using the maximum tolerable dose of antiarrhythmic drugs, including metoprolol and amiodarone, the patient still presented with recurrent symptoms and heart failure. Catheter ablation of AF might help the patient restore normal sinus rhythm and improve the patient's quality of life; however, considering the diffuse calcification, the operation was of great difficulty and risk, as there was no previous report on catheter ablation in patients with myocardial calcification for reference. We proposed the use of intracardiac echocardiography (ICE) during the procedure, but the patient refused for financial reasons. Atrial septal puncture guided by fluoroscopy became more difficult due to the calcification of the atrial septa. Electroanatomic mapping of the atrium was performed with a Pentaray catheter on the CARTO 3 system (Biosense Webster), which showed areas of low voltage (bipolar voltage amplitude ≤1.5 mV) in the region of calcification (Figure 2D). Persistent AF was terminated after circumferential pulmonary vein isolation and electrical posterior box isolation, and no further ablation was performed (Figure 2E). Fortunately, persistent AF was converted to sinus rhythm, and there was no recurrence was observed during the one-year follow-up (Figures 2F, G).

## 3. Discussion

Cases of myocardial calcification, especially those associated with AF and heart failure, are rare. It is well recognized that myocardial calcification can reduce the contractility of the myocardium, as well as disturb the cardiac conduction system, causing heart failure and arrhythmias. To our knowledge, this is the first report to focus on the electrophysiological aspects of cardiac arrhythmias in a patient with diffuse myocardial calcification from a clinical perspective.

Previous reports have suggested that calcium deposition might involve the atrioventricular node and conduction system in patients with chronic renal failure (5, 6). However, this patient had no obvious cardiac conduction defects, with only areas of low voltage in electroanatomic mapping associated with calcification. A previous study showed that mitral annular calcification is associated with AF (7). In addition, mitral annular calcification might also be associated with an increased risk of embolization (8, 9). The relationship between myocardial calcification and AF, as well as its underlying mechanism, is still poorly understood. We speculated that diseases associated with calcification itself, such as chronic renal failure, myocardial infarction, and hyperparathyroidism, might be risk factors for AF (10). In addition, left atrial enlargement and pressure overload caused by the calcification of the myocardium and valves might also be a medium for the association between myocardial calcification and AF (11).

To our knowledge, there have been no previous reports on catheter ablation for AF in patients with myocardial calcification. It could be speculated that patients with myocardial calcification are more susceptible to tachycardia-induced heart failure due to their reduced systolic and diastolic dysfunction, as well as functional impairment of the valves. Catheter ablation might be a reasonably effective procedure to maintain the sinus rhythm; however, relevant clinical experience in patients with myocardial calcification is still lacking. Histological examination showed that the calcified areas were surrounded by fibrotic tissue and contained large fibrin deposition (12). The first step of the ablation strategy for the patient was pulmonary vein isolation. Considering that the posterior wall may play a potential role in the development and maintenance of AF (13), posterior wall isolation was also performed. However, the areas of low voltage in the region of calcification, especially in the left atrium, as shown in the 3-dimensional electroanatomic mapping, should be considered and avoided during the ablation process. Although calcification might not be a trigger for arrhythmias, it may still play an important role in the maintenance of AF; however, ablation of the region of calcification might be ineffective and dangerous. For instance, the extensive calcifications may penetrate the endocardial surface and pose a potential risk of embolization during catheter manipulation. Cerebral protection devices may be useful for preventing such iatrogenic periprocedural stroke during ablation (14). In our opinion, ICE should be used in patients with diffuse calcification to guide transseptal puncture and ablation procedures. Firstly, ICE can help locate the needle tip position precisely and avoid the calcified regions of the atrial septa during the process of atrial septal puncture. Furthermore, ICE provides real-time visualization of cardiac structures during ablation procedures, thereby avoiding the ablation in calcified regions, especially those with endocardial calcification, which might prevent potential calcific embolization (15).

## 4. Conclusion

Catheter ablation is challenging in patients with myocardial calcification. Despite the lack of clinical experience, it is still worth performing when there are expected benefits. Ablation is also one of the best treatments for such patients.

## Data Availability

The original contributions presented in the study are included in the article/Supplementary Material, further inquiries can be directed to the corresponding authors.
